# A case report of Wernicke Korsakoff syndrome in a patient with cholangiocellular carcinoma: An underestimated cause of encephalopathy in cancer patients

**DOI:** 10.1097/MD.0000000000031904

**Published:** 2022-12-02

**Authors:** Oguzhan Koca, Bilal Demir, Sumeyra Derin, Zeynep Hande Turna

**Affiliations:** a Department of Internal Medicine, Cerrahpasa Medical Faculty, Istanbul University-Cerrahpasa, Istanbul, Turkey; b Department of Radiology, Cerrahpasa Medical Faculty, Istanbul University-Cerrahpasa, Istanbul, Turkey; c Division of Medical Oncology, Department of Internal Medicine, Cerrahpasa Medical Faculty, Istanbul University-Cerrahpasa, Istanbul, Turkey.

**Keywords:** case report, cholangiocellular carcinoma, thiamine replacement, total parenteral nutrition, Wernicke Korsakoff syndrome

## Abstract

Wernicke’s encephalopathy, a disorder caused by thiamin deficiency, is characterized by a classical triad of encephalopathy, ataxia, and ophthalmoplegia. Although alcoholism is the most common predisposing factor, it can also be associated with nonalcoholic states (hyperemesis gravidarum, intestinal obstruction, bariatric surgery, and others). This work presents a case of nonalcoholic Wernicke-Korsakoff syndrome diagnosed in a cholangiocellular carcinoma patient and literature review.

**Case report:**

A 65-year-old male patient with a history of cholangiocellular carcinoma (Klatskin tumor) was treated with radiotherapy at the operation site after Roux-en-Y hepaticojejunostomy. During follow-up, the patient developed gastric outlet obstruction and was diagnosed with peritoneal carcinomatosis after a palliative gastrojejunostomy. As the patient could not tolerate oral nutrition during hospitalization, total parenteral nutrition was administered. After 10 days of admission, the patient showed decreased response to verbal stimuli as well as bilateral horizontal nystagmus, lethargy, and disorientation. Furthermore, the patient displayed confabulation. Clinical and imaging findings were consistent with Wernicke’s encephalopathy. Therefore, treatment with intravenous thiamin replacement was initiated. The patient’s encephalopathy regressed on the second day after treatment, and he recovered the place-person-time orientation. In the following month, the abnormal imaging findings were almost entirely resolved.

**Conclusion:**

In order to prevent irreversible brain damage induced by chronic thiamin deficiency, thiamin replacement therapy with parenteral nutrition solutions should be included as a treatment for hospitalized cancer patients unable to receive enteral nutrition for a long time.

## 1. Introduction

Wernicke’s encephalopathy is a disorder caused by thiamin deficiency typically presented as a triad of encephalopathy, ataxia, and ophthalmoplegia. However, in clinical settings, only one-third of the patients manifest these 3 classic symptoms.^[1]^ Approximately 80% of patients with untreated Wernicke’s encephalopathy develop Korsakoff syndrome, characterized by confabulation, anterograde and retrograde amnesia.^[2]^ The most common predisposing factor is alcoholism, but it can also be associated with nonalcoholic states such as hyperemesis gravidarum, intestinal obstruction, bariatric surgery, malignancy, chemotherapy, and hemodialysis.^[3]^ Herein, we present a nonalcoholic Wernicke-Korsakoff syndrome case in a patient with cholangiocellular carcinoma.

## 2. Case report

A 65-year-old male patient with cholangiocellular carcinoma (Klatskin tumor) and a negative history of alcohol consumption was treated with Roux-en-Y hepaticojejunostomy, followed by radiotherapy. No metastatic lesions were detected at the time of initial diagnosis. However, after a 1-year follow-up from the first surgery, the patient developed gastric outlet obstruction and underwent palliative gastrojejunostomy, leading to the detection of peritoneal carcinomatosis. This malignancy was diagnosed as metastatic cholangiocellular carcinoma, and chemotherapy (gemcitabine) was initiated.

The patient was administered total parenteral nutrition for 2 months following surgery because of his inability to tolerate oral nutrition. After the first course of chemotherapy, the patient was admitted to the hospital with fever, and initiated parenteral antibiotic therapy. During hospitalization, the patient developed persistent nausea and vomiting due to subileus caused by compression from the intraperitoneal tumor implants. As the patient was still intolerant to oral nutrition, total parenteral nutrition was continued during hospitalization.

**Figure 1.**
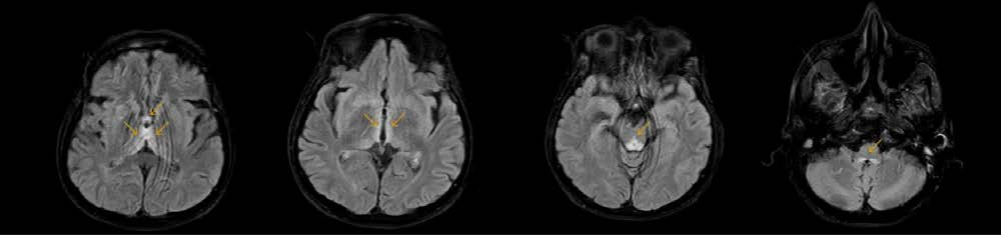
Diffusion-weighted MRI images showing hyperintensities compatible with vasogenic edema observed in the bilateral medial thalamus, mamillary bodies, and dorsal brain stem of the patient in the FLAIR sequences. FLAIR = fluid attenuated inversion recovery, MRI = magnetic resonance imaging.

On the tenth day of hospitalization, the patient exhibited a decreased response to verbal stimuli. Diffusion-weighted brain magnetic resonance imaging was conducted, which revealed increased symmetrical fluid attenuated inversion recovery signal intensities in the bilateral mammillary bodies, medial aspects of the thalamus, walls of the third ventricle, periaqueductal gray matter, and dorsal brain stem (Fig. 1). These findings were consistent with those of Wernicke’s encephalopathy. In addition, the patient was lethargic, disoriented, presented bilaterally horizontal nystagmus, and displayed confabulation. Gait ataxia could not be evaluated as the patient was immobile and presented low performance status.

**Figure 2.**
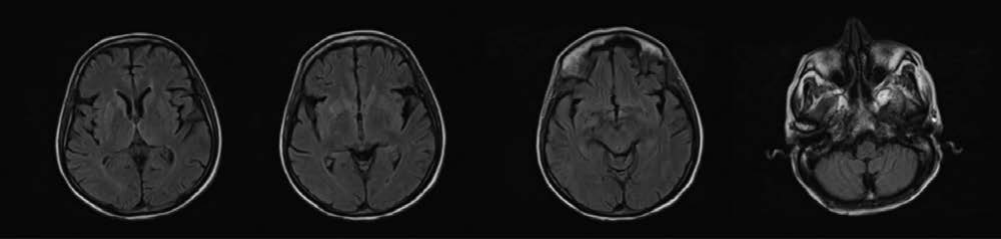
FLAIR MRI imaging shows that previous hyperintensities were almost completely resolved 1 month after thiamin replacement. FLAIR = fluid attenuated inversion recovery, MRI = magnetic resonance imaging.

Intravenous thiamin replacement therapy was started at 200 mg 3 times a day. The patient’s encephalopathy regressed on the second day after treatment, and his place-person-time orientation returned. However, the nystagmus persisted. After 1 month of thiamin replacement, the plasma thiamin level was 301.9 µg/L (reference range: 25–75 µg/L), and the previously noted fluid attenuated inversion recovery hyperintensities were almost completely resolved in the first-month follow-up diffusion magnetic resonance imaging (Fig. 2). Thiamine replacement therapy (100 mg/day) was maintained since the precipitating factors of Wernicke’s encephalopathy were still present.

## 3. Discussion

Reports on Wernicke’s encephalopathy in patients who had previously undergone gastrointestinal cancer surgery or received chemotherapy are not uncommon in the literature. The primary malignancies documented in these studies were colorectal, gastric, pancreatic, and gallbladder cancer, pelvic sarcoma, acute myelogenous leukemia, inflammatory breast carcinoma, and multiple myeloma.^[4–20]^ To the best of our knowledge the present case is the first to report a Wernicke’s encephalopathy in a patient diagnosed with cholangiocellular carcinoma. Previous case reports of Wernicke’s encephalopathy in cancer patients after gastrointestinal surgery are listed in Table 1. The encephalopathy’s common etiopathogenesis in these patients, regardless of cancer type, can be explained by the inability to maintain oral-enteral nutrition and thiamin absorption after surgical excision of portions of the gastrointestinal tract. Other risk factors reported in the literature include malnutrition, increased thiamin consumption due to rapidly growing tumors, appetite loss, use of chemotherapeutic agents, nausea, and vomiting.^[14]^

**Table 1:** Case reports on Wernicke’s encephalopathy in cancer patients after gastrointestinal surgery.

Author	Year	Number of cases	Cancer type	Surgery type
Batori et al^[5]^	1995	1	Gastric cancer	Subtotal gastrectomy
Arai et al^[6]^	1997	2	Gastric cancer	Total gastrectomy
Shimomura et al^[7]^	1998	3	Gastric cancer	Total gastrectomy, subtotal gastrectomy
Attard et al^[8]^	2006	1	Benign gastric tumor**	Subtotal gastrectomy
Karayiannakis et al^[9]^	2011	1	Pancreatic cancer	Pancreaticoduodenectomy
Rufa et al^[10]^	2011	10	Pancreatic cancer, colon cancer, gallbladder cancer, gallbladder adenoma	Abdominal-perineal resection, gastro-entero-anastomosis, hemicolectomy, pancreaticoduodenectomy, cholecystectomy
Busani et al^[11]^	2014	1	Duodenal cancer	Pancreaticoduodenectomy
Kilinc et al^[12]^	2015	1*	Pancreatic cancer	Pancreaticoduodenectomy
Wu et al^[13]^	2016	1*	Pancreatic cancer	Pancreaticoduodenectomy
Restivo et al^[14]^	2016	19	Gastric cancer, colorectal cancer	Total/distal gastrectomy, hemicolectomy, anterior resection, abdominal perineal resection
Tozzo et al^[15]^	2017	1	Gastric cancer	Subtotal gastrectomy
Tsao et al^[16]^	2017	1*	Gastric cancer	Subtotal gastrectomy
AbdelRazek et al^[17]^	2018	1	Pancreatic cancer	Pancreaticoduodenectomy
Kim et al^[18]^	2019	1	Ampulla of vater cancer	Pancreaticoduodenectomy
Fedeli et al^[19]^	2020	2	Gastric cancer, colorectal cancer	Subtotal gastrectomy, anterior resection of rectum
Li et al^[20]^	2021	1	Colon cancer	Hemicolectomy
Present case	2021	1	Cholangiocelular carcinoma (Klatskin tumor)	Roux-en-Y hepaticojejunostomy, gastrojejunostomy

* Two cases were reported in each of these publications, but only 1 of the 2 patients had undergone surgery for cancer.

** Surgery was performed for a non-cancerous gastric tumor.

Prompt diagnosis of Wernicke’s encephalopathy is crucial to prevent the progression to Korsakoff syndrome. In 2017, a multicenter study on Wernicke’s encephalopathy patients determined that the classical triad of symptoms for the condition was observed in only one-third of the cases.^[1]^ In the event of clinical suspicion, thiamin replacement therapy should be immediately started without waiting for the thiamin level test result. For instance, even though we could not determine the patient’s plasma thiamin level at the time of diagnosis in the current case, the treatment was administered since the patient’s clinical and radiological status was consistent with Wernicke-Korsakoff syndrome.

In hospitalized cancer patients with reduced oral intake, administration of intravenous glucose solutions can accelerate the consumption of thiamin due to increased glycolysis, which may induce acute thiamin deficiency. This acute deficiency particularly presents a high risk in patients who have undergone gastrointestinal surgery. Prophylactic thiamin replacement before or at the time of parenteral nutrition should be considered a preventive treatment. Therefore, in the case of confusional state in cancer patients, Wernicke’s encephalopathy should also be considered as an etiology in addition to the other common such as infection, metastasis, and cerebrovascular events.

A retrospective review article from 2021 examined data from a total of 586 patients with nonalcoholic Wernicke’s encephalopathy and determined that 22% were cancer patients. This study also stated that low doses of thiamin cause chronic Wernicke-Korsakoff syndrome and recommended parenteral thiamin administration of 500 mg, 3 times per day as a treatment for adult patients.^[21]^

A review from 2012 (Sriram et al, American Society for Parenteral and Enteral Nutrition) defined the risk groups for thiamin deficiency. Based on their clinical experience and practice, the authors recommended thiamin administration under certain conditions, one of these being patients at risk of refeeding syndrome (cachexia, malignancies, and chronic malnutrition). The thiamin dosage recommendation for a patient at risk of deficiency is 100 mg, and 200 mg for a patient with proven deficiency, in both cases, administered parenterally 3 times a day.^[22]^ The European Federation of the Neurological Societies guidelines for diagnosis, therapy, and prevention of Wernicke’s encephalopathy, recommend prophylactic parenteral administration of 200 mg thiamin before carbohydrates for all patients admitted to the emergency room.^[23]^ The National Comprehensive Cancer Network guidelines for gastric cancer (version 2.2022) recommend monitoring vitamin B12 and iron levels in total or subtotal gastrectomy survivors. However, there is still no recommendation for thiamin level monitoring.^[24]^

## 4. Conclusion

In order to prevent irreversible brain damage due to untreated Wernicke’s encephalopathy, thiamin replacement therapy administered along with dextrose fluid or parenteral nutrition solutions should be considered as prophylactic treatment in hospitalized cancer patients where enteral nutrition cannot be provided for an extended period.

## Author contributions

**Conceptualization:** Oguzhan Koca, Sumeyra Derin, Zeynep Hande Turna.

**Formal analysis:** Sumeyra Derin, Zeynep Hande Turna.

**Investigation:** Oguzhan Koca.

**Software:** Bilal Demir.

**Supervision:** Zeynep Hande Turna.

**Visualization:** Bilal Demir.

**Writing – original draft:** Oguzhan Koca.

**Writing – review & editing:** Oguzhan Koca, Sumeyra Derin, Zeynep Hande Turna.
